# Supplementary figures and images for: Specialisation of meiotic kinetochores revealed through a synthetic spindle assembly checkpoint strategy
Source: eLife. 2026 Apr 9;15:RP110117. doi: 10.7554/eLife.110117 (PMC13065327; doi:10.7554/eLife.110117)

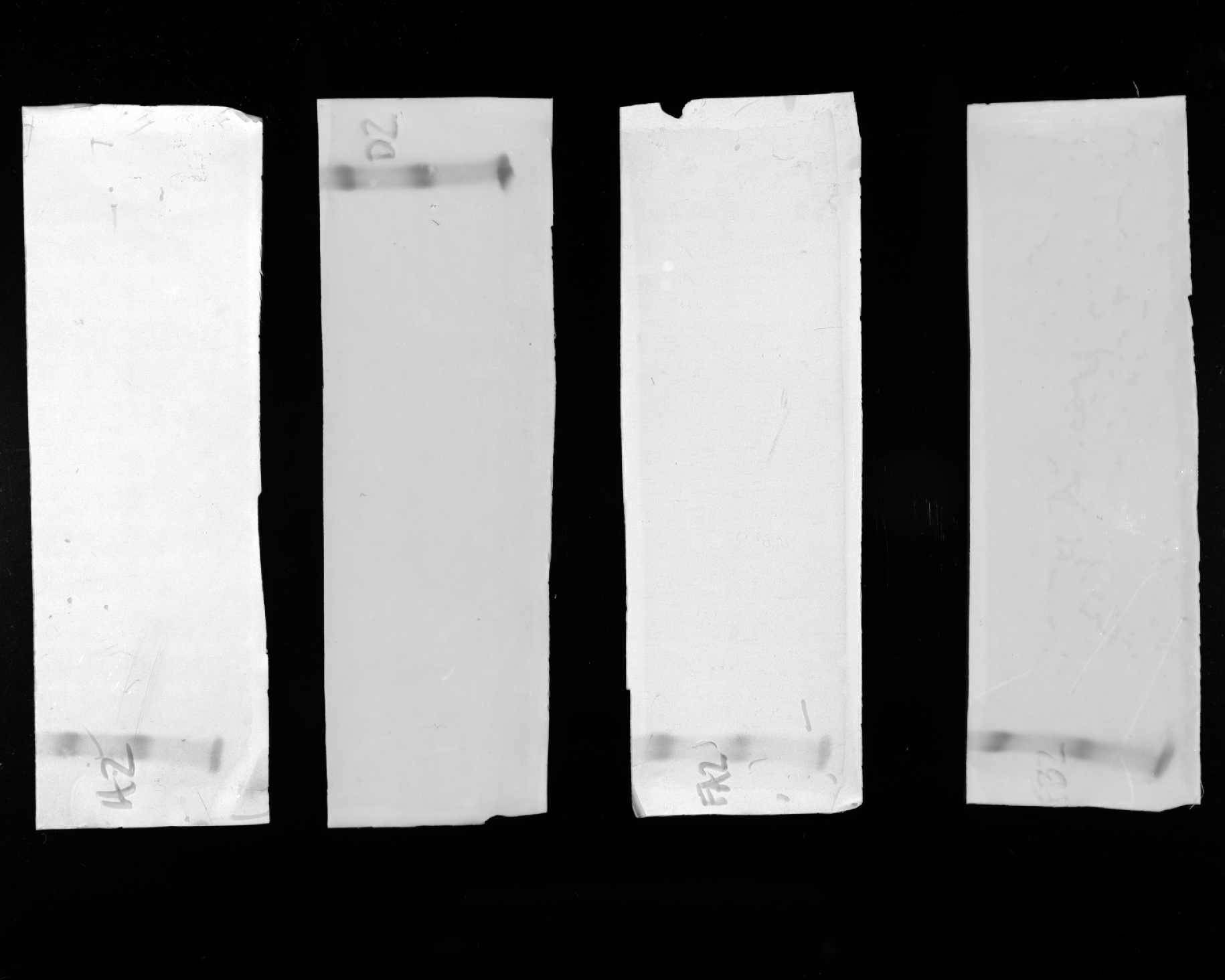

Supplement: Figure 3—source data 1. [file elife-110117-fig3-data1.zip › Figure1-source data 1/Pgk1_Colorimetric.tif]

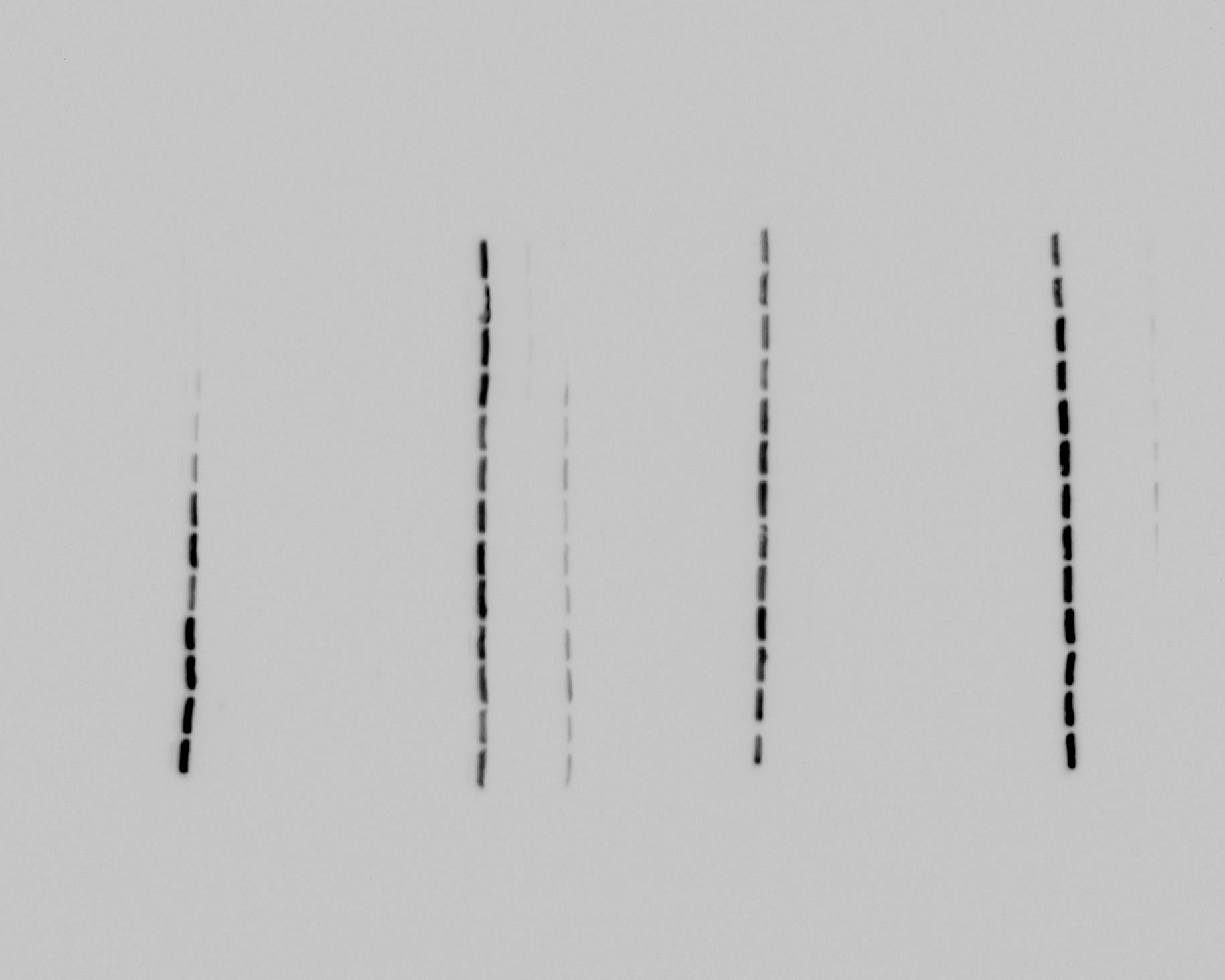

Supplement: Figure 3—source data 1. [file elife-110117-fig3-data1.zip › Figure1-source data 1/Myc_WB.tif]

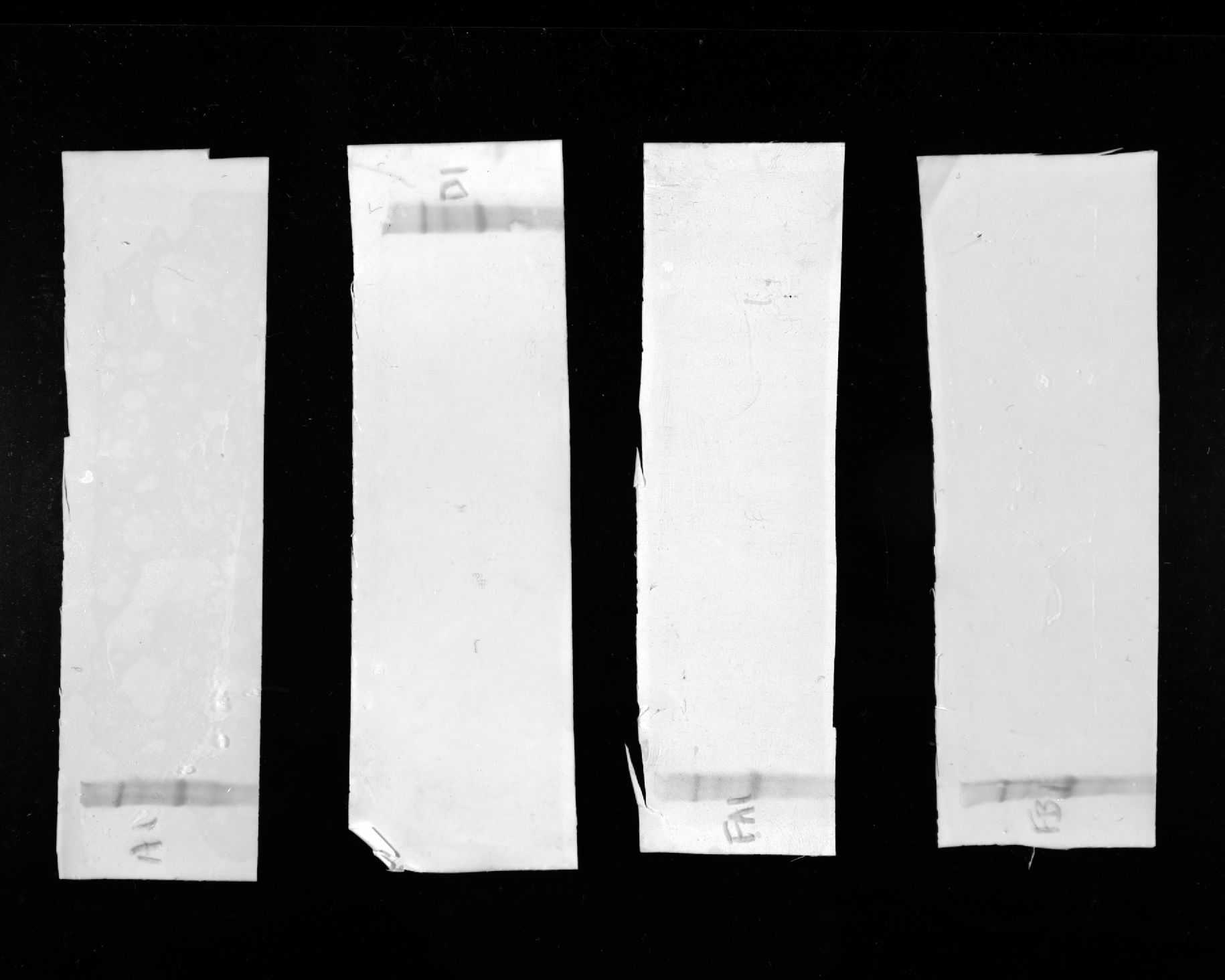

Supplement: Figure 3—source data 1. [file elife-110117-fig3-data1.zip › Figure1-source data 1/Myc_Colorimetric.tif]

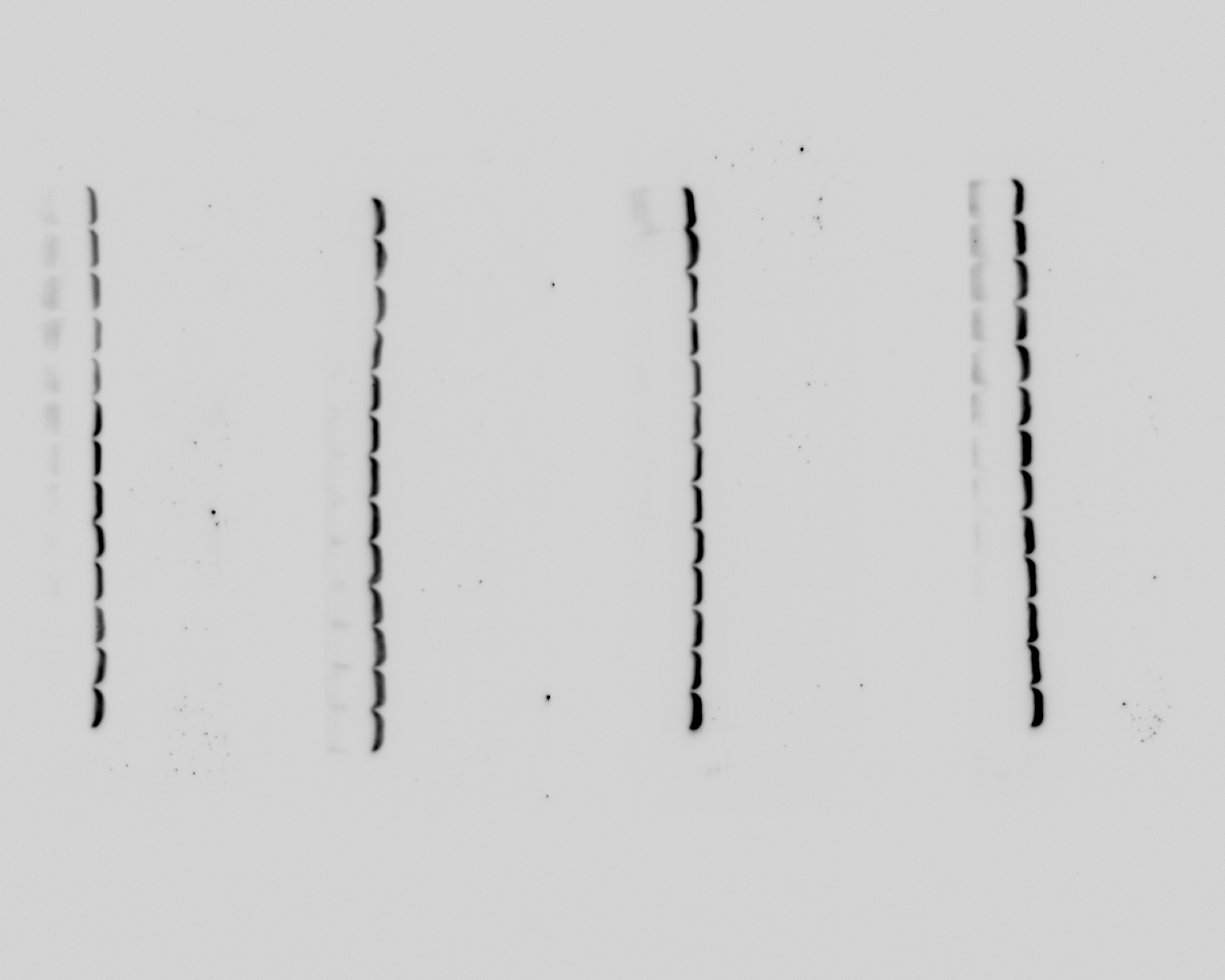

Supplement: Figure 3—source data 1. [file elife-110117-fig3-data1.zip › Figure1-source data 1/Pgk1_WB.tif]

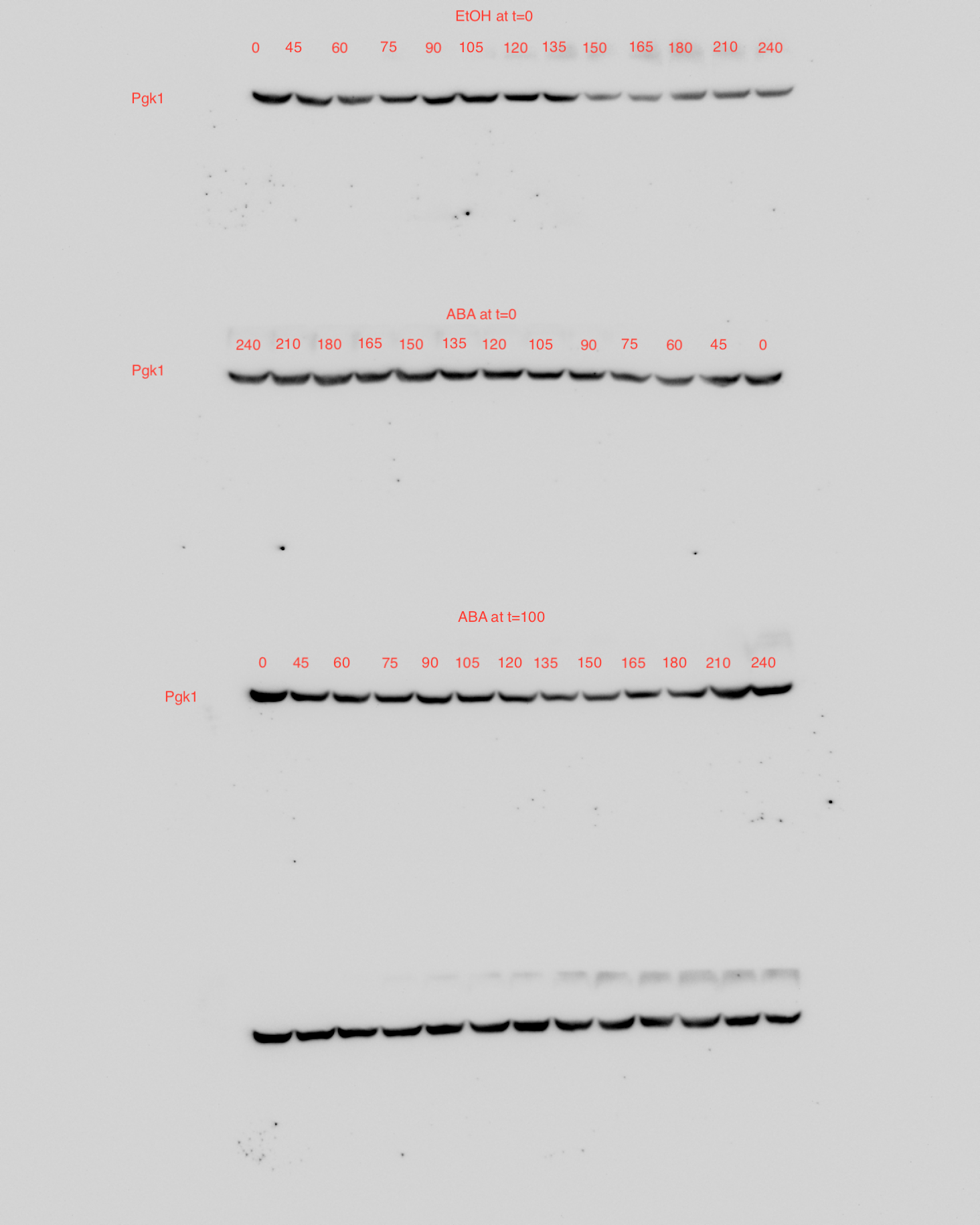

Supplement: Figure 3—source data 2. [file elife-110117-fig3-data2.zip › Figure1-source data 2/Pgk1_WB_annotated.tif]

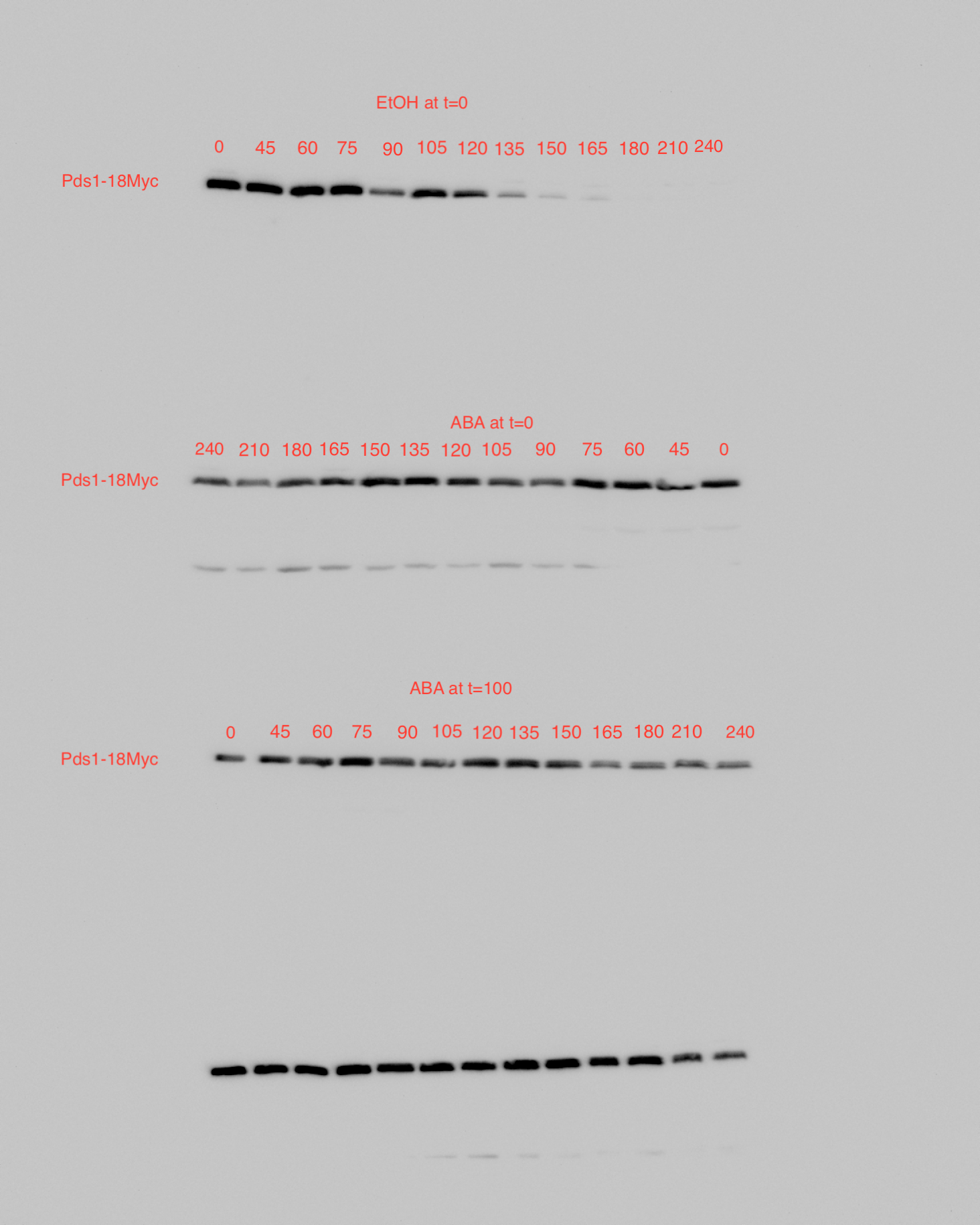

Supplement: Figure 3—source data 2. [file elife-110117-fig3-data2.zip › Figure1-source data 2/Myc_WB_annotated.tiff]

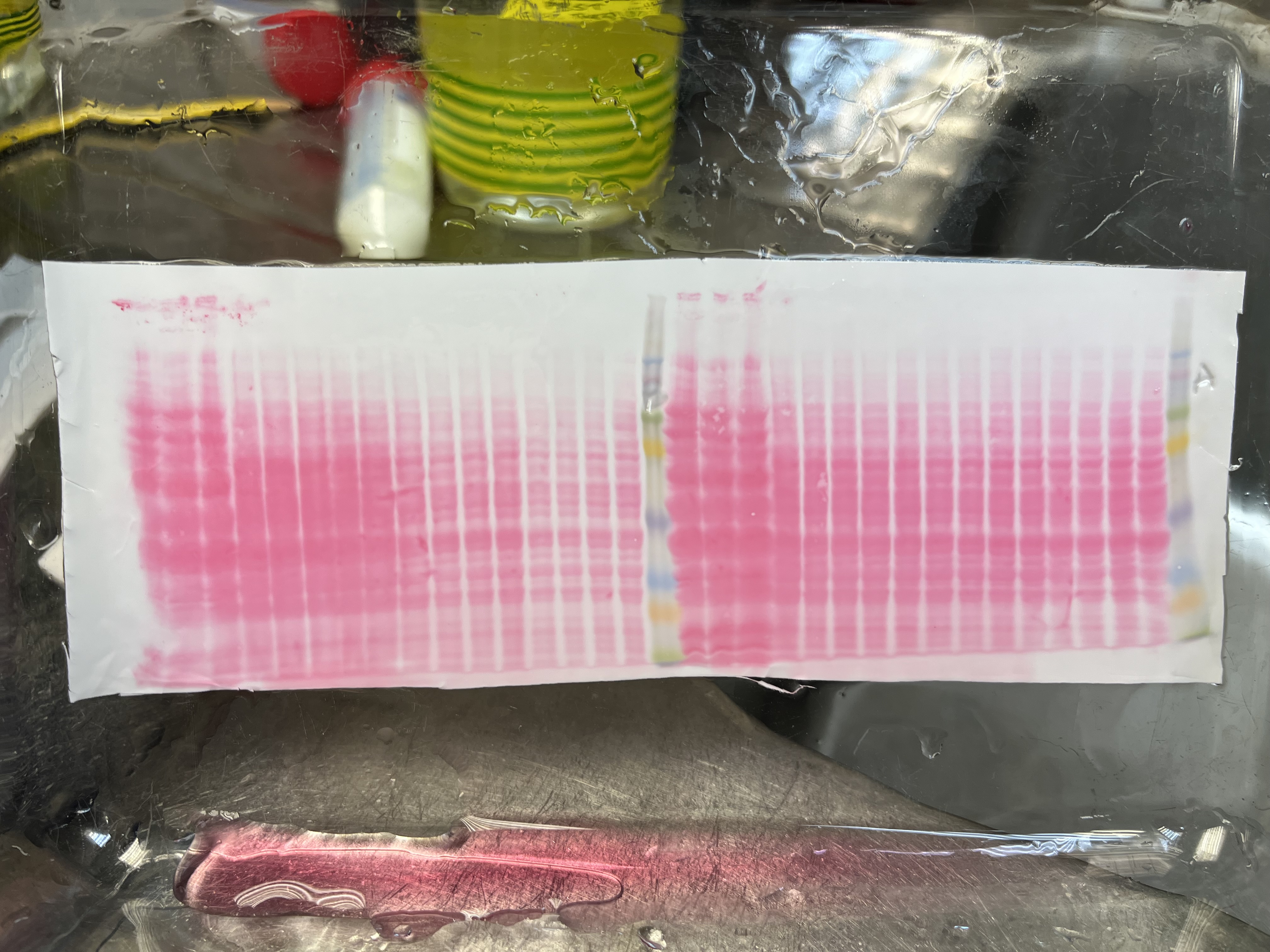

Supplement: Figure 3—figure supplement 1—source data 1. [file elife-110117-fig3-figsupp1-data1.zip › Figure3-figure supplement 1-source data 1/D/ponceau.jpg]

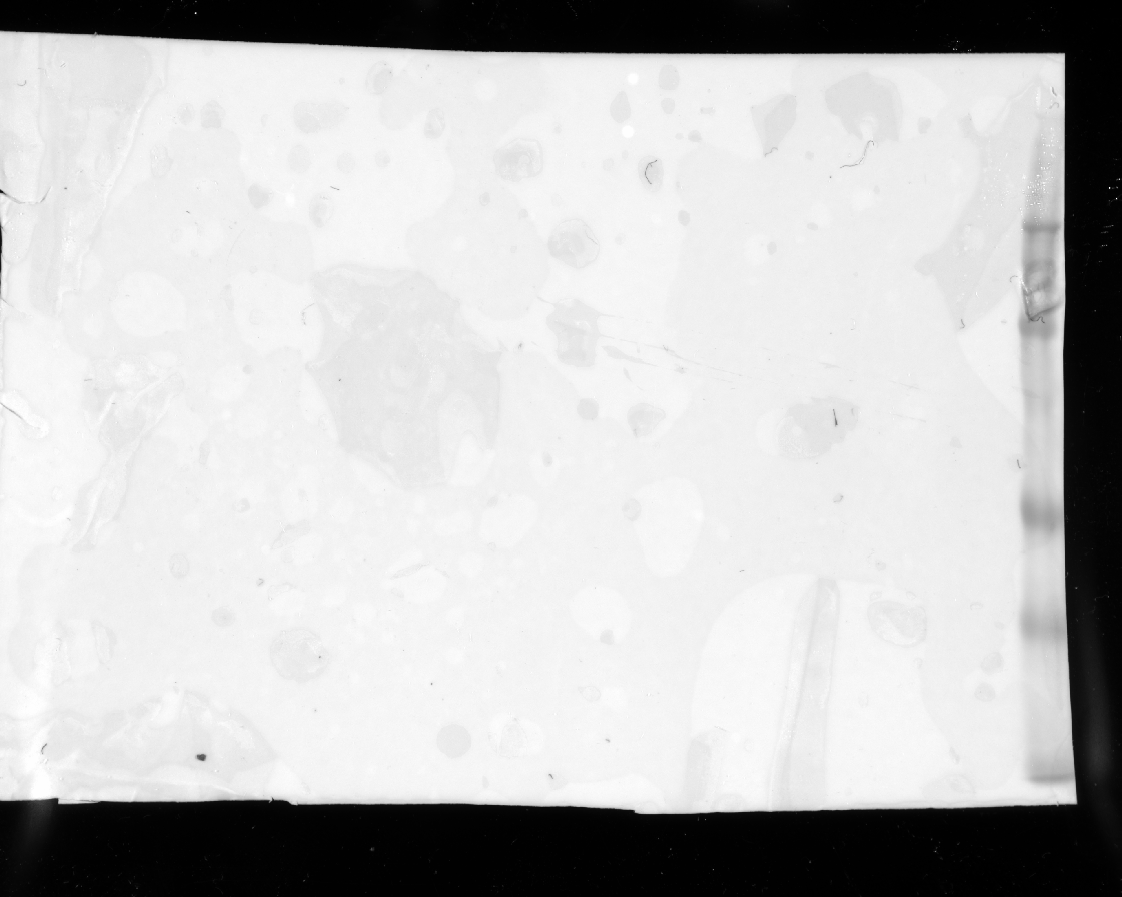

Supplement: Figure 3—figure supplement 1—source data 1. [file elife-110117-fig3-figsupp1-data1.zip › Figure3-figure supplement 1-source data 1/D/Pgk1(Colorimetric).tif]

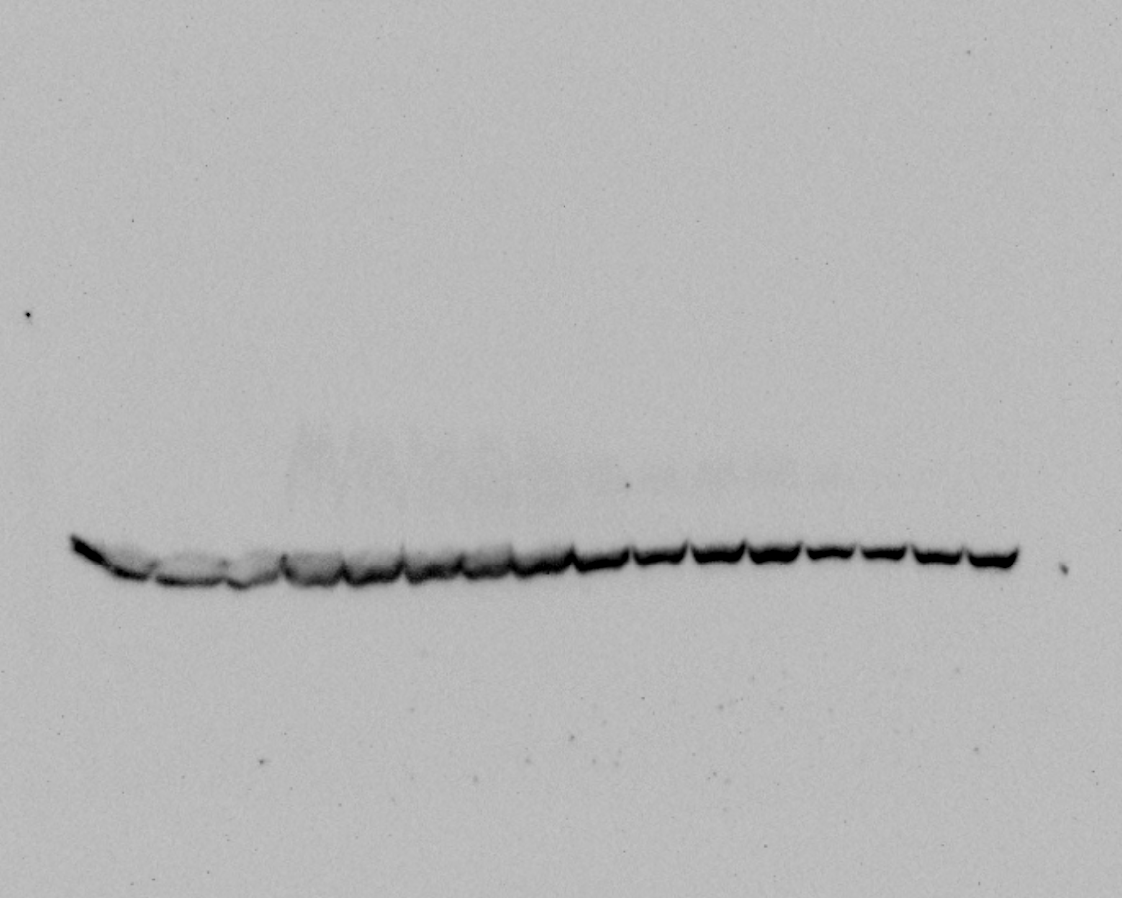

Supplement: Figure 3—figure supplement 1—source data 1. [file elife-110117-fig3-figsupp1-data1.zip › Figure3-figure supplement 1-source data 1/D/Pgk1(Chemiluminescence).tif]

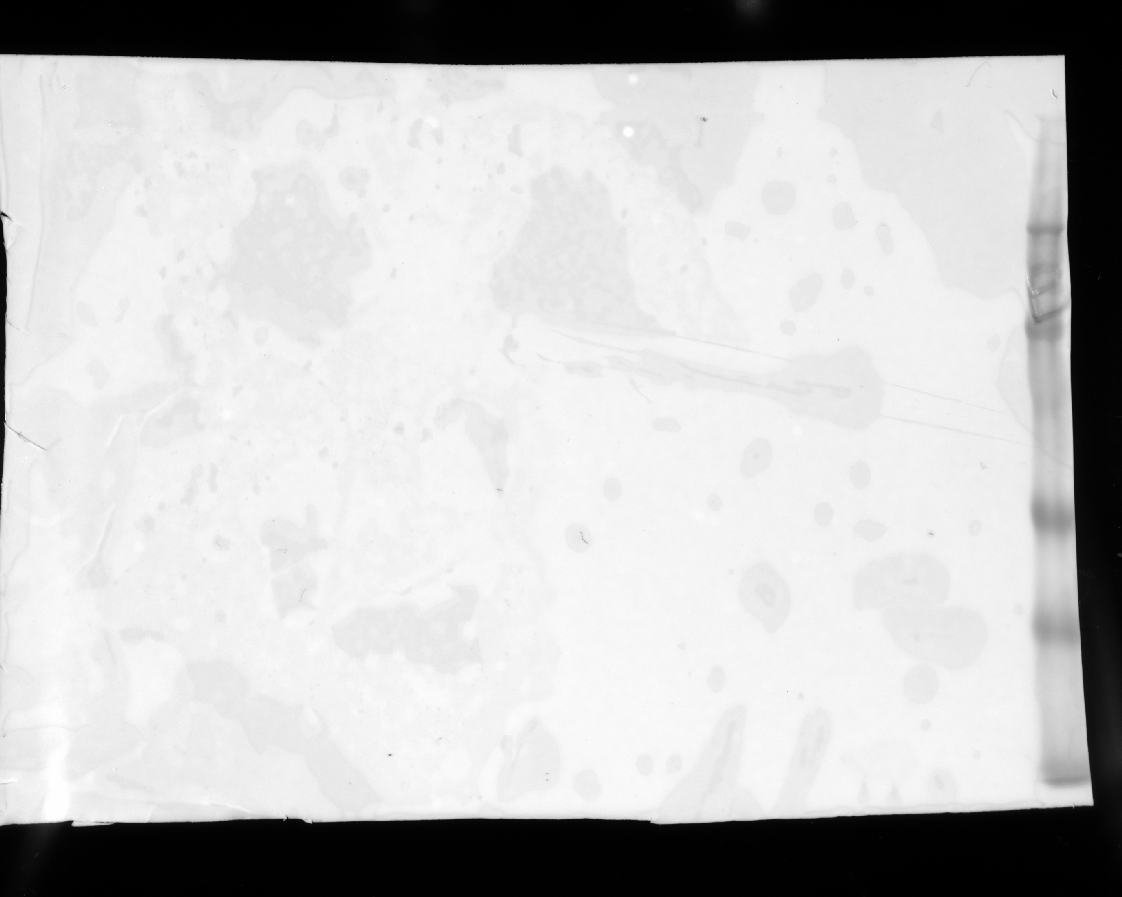

Supplement: Figure 3—figure supplement 1—source data 1. [file elife-110117-fig3-figsupp1-data1.zip › Figure3-figure supplement 1-source data 1/D/FLAG(Colorimetric).tif]

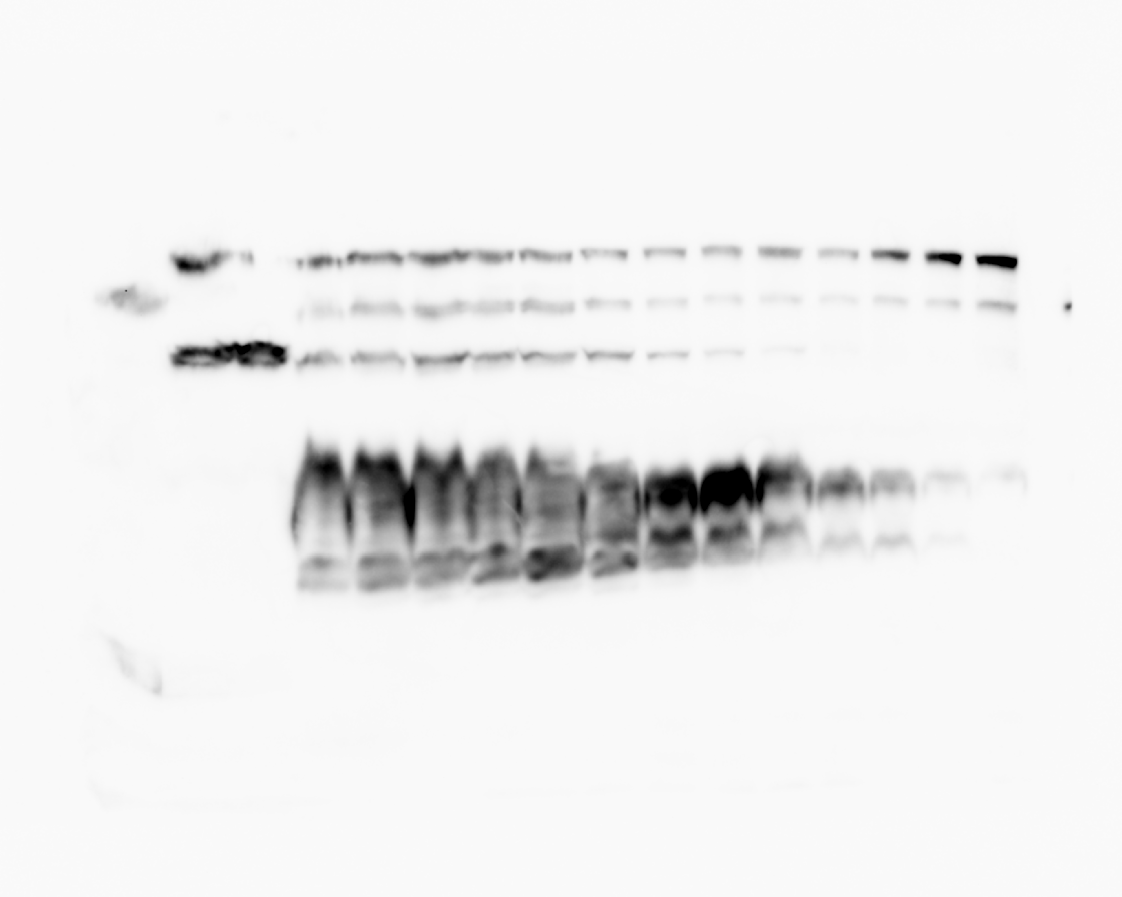

Supplement: Figure 3—figure supplement 1—source data 1. [file elife-110117-fig3-figsupp1-data1.zip › Figure3-figure supplement 1-source data 1/D/FLAG(Chemiluminescence).tif]

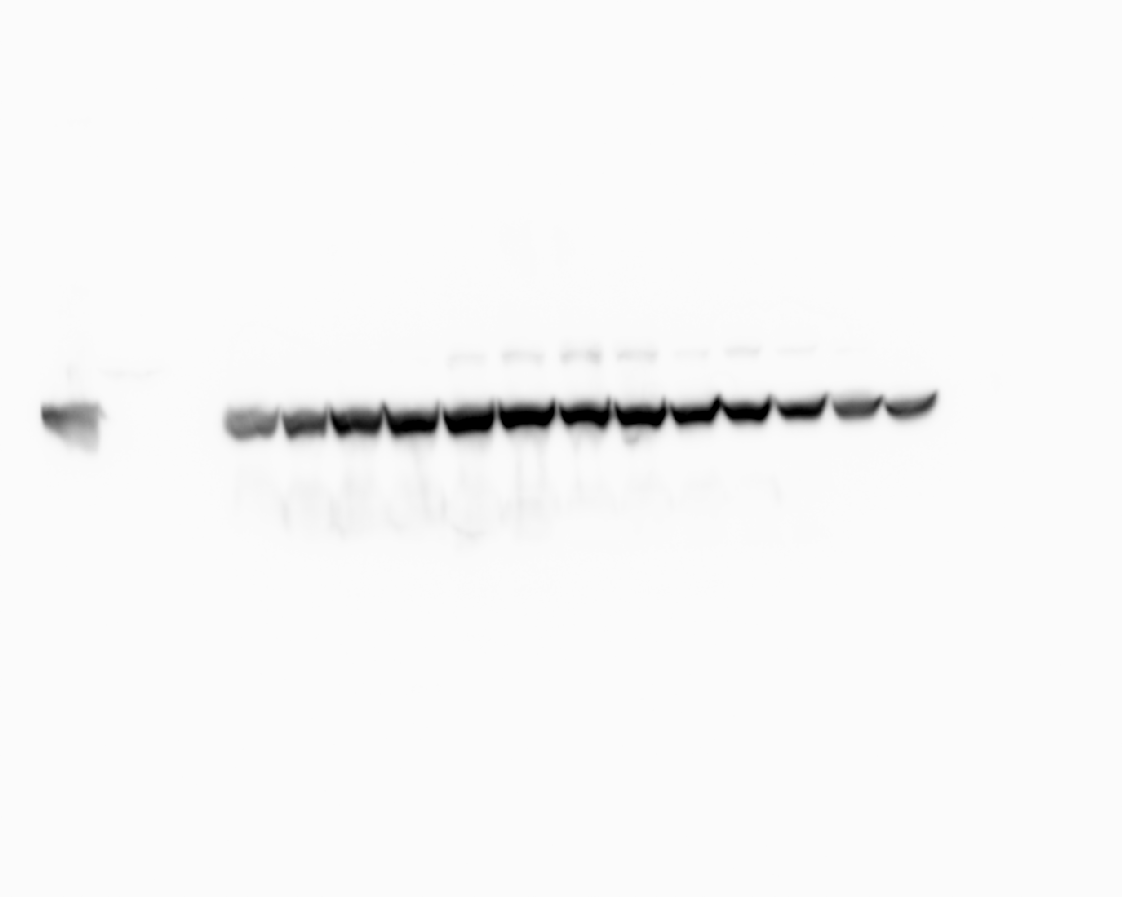

Supplement: Figure 3—figure supplement 1—source data 1. [file elife-110117-fig3-figsupp1-data1.zip › Figure3-figure supplement 1-source data 1/B/V5_short.tif]

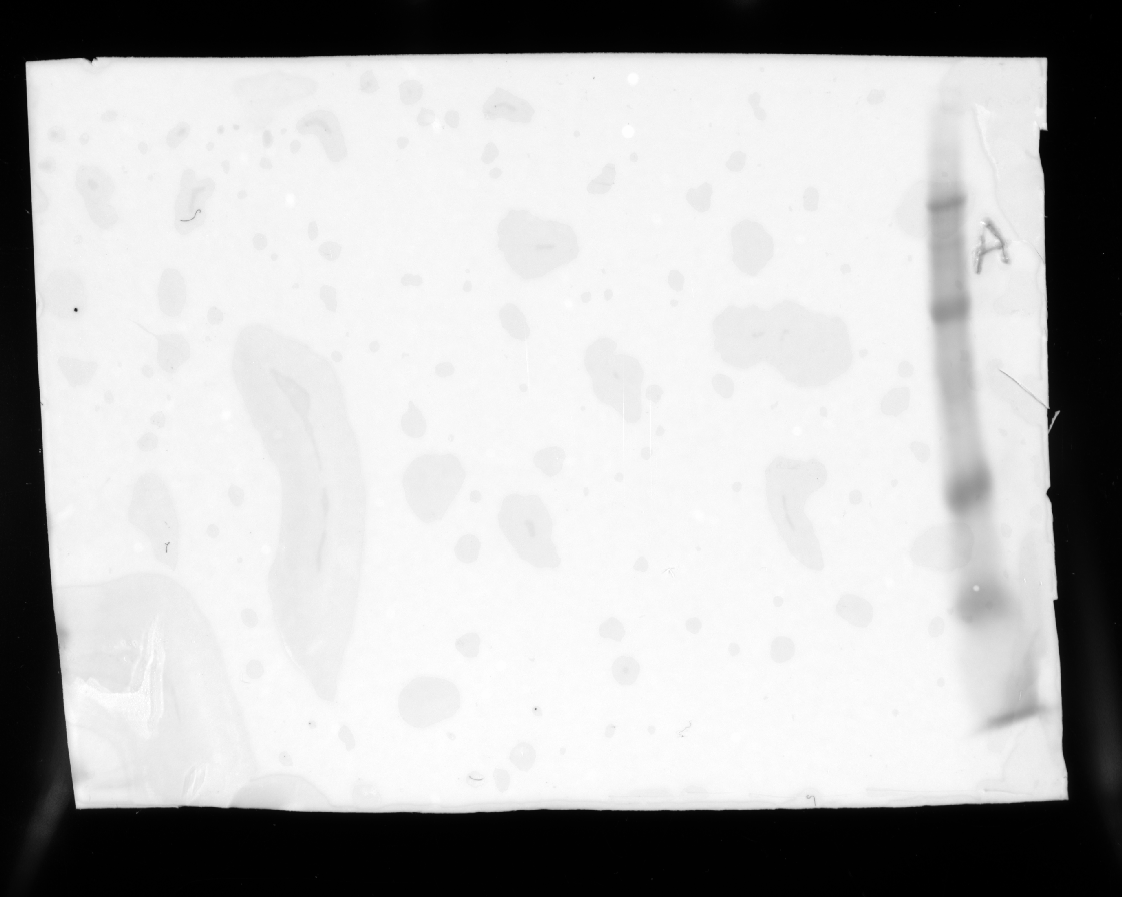

Supplement: Figure 3—figure supplement 1—source data 1. [file elife-110117-fig3-figsupp1-data1.zip › Figure3-figure supplement 1-source data 1/B/V5(Colorimetric)_for_reference.tif]

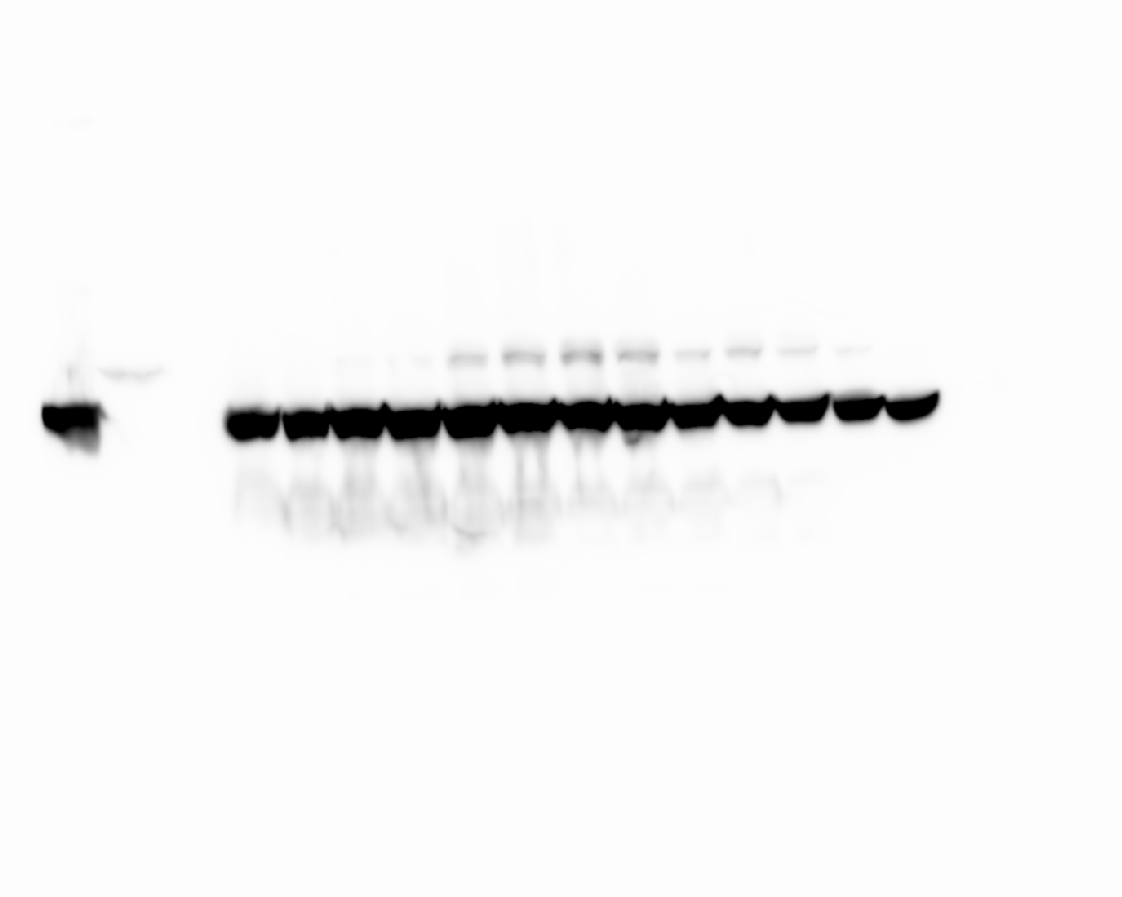

Supplement: Figure 3—figure supplement 1—source data 1. [file elife-110117-fig3-figsupp1-data1.zip › Figure3-figure supplement 1-source data 1/B/V5_long.tif]

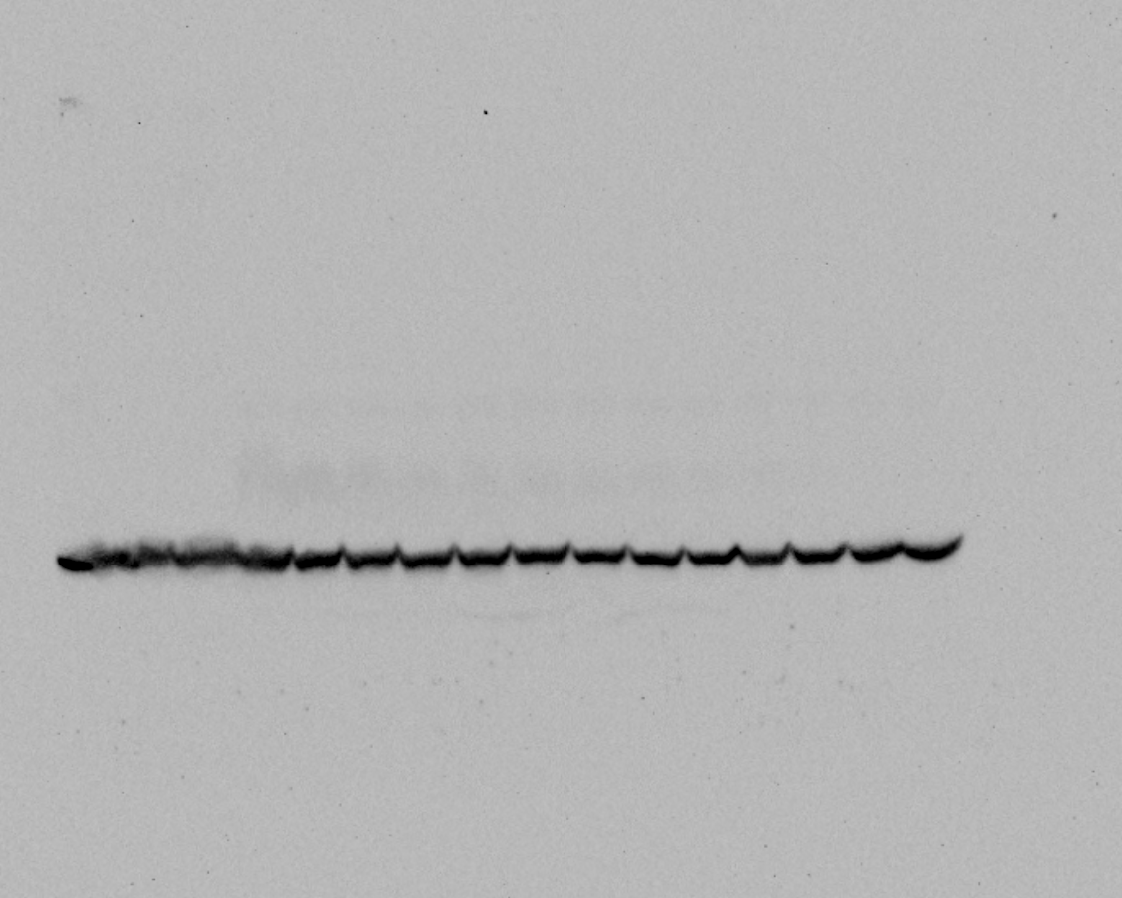

Supplement: Figure 3—figure supplement 1—source data 1. [file elife-110117-fig3-figsupp1-data1.zip › Figure3-figure supplement 1-source data 1/B/Pgk1(Chemiluminescence)_for_reference.tif]

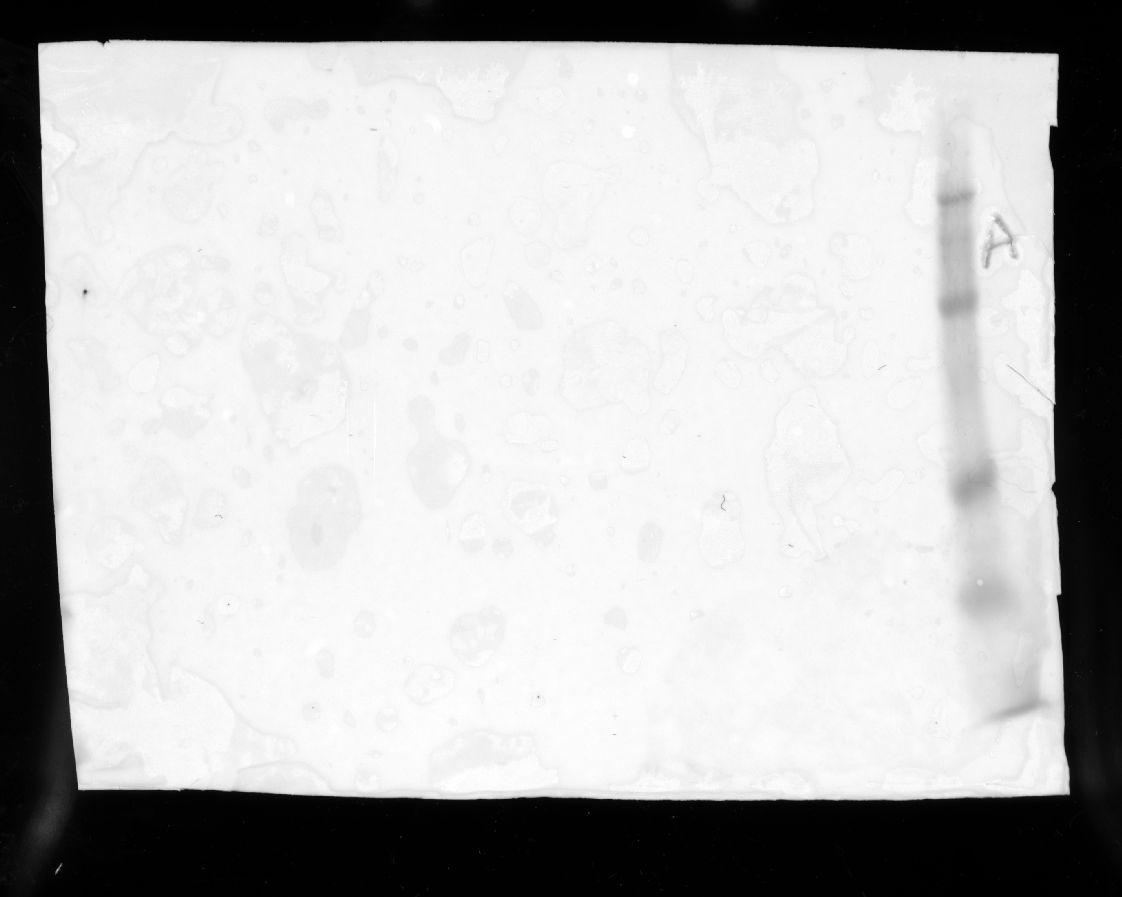

Supplement: Figure 3—figure supplement 1—source data 1. [file elife-110117-fig3-figsupp1-data1.zip › Figure3-figure supplement 1-source data 1/B/Pgk1.tif]

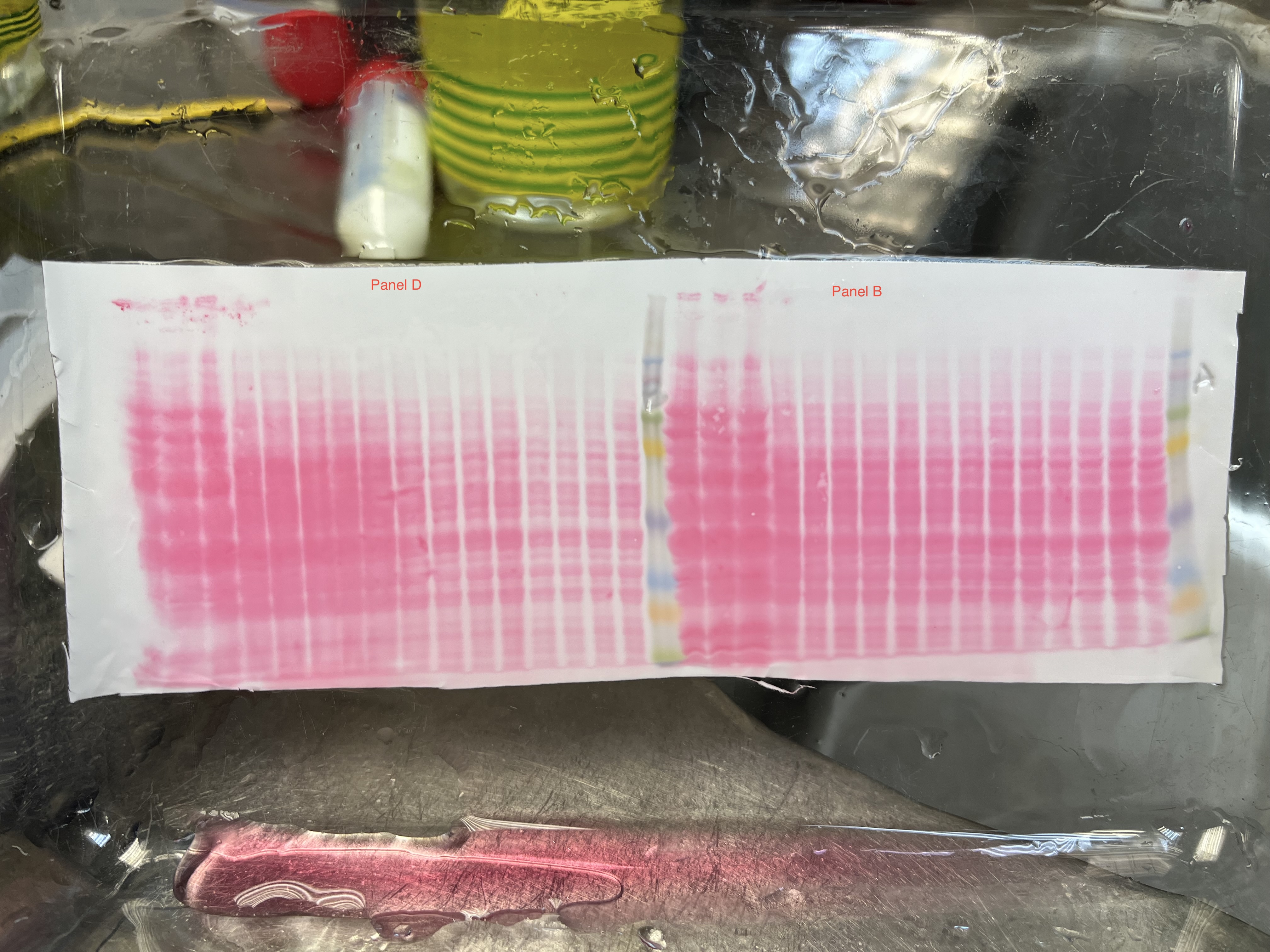

Supplement: Figure 3—figure supplement 1—source data 2. [file elife-110117-fig3-figsupp1-data2.zip › Figure3-figure supplement 1-source data 2/D/ponceau_annotated.jpg]

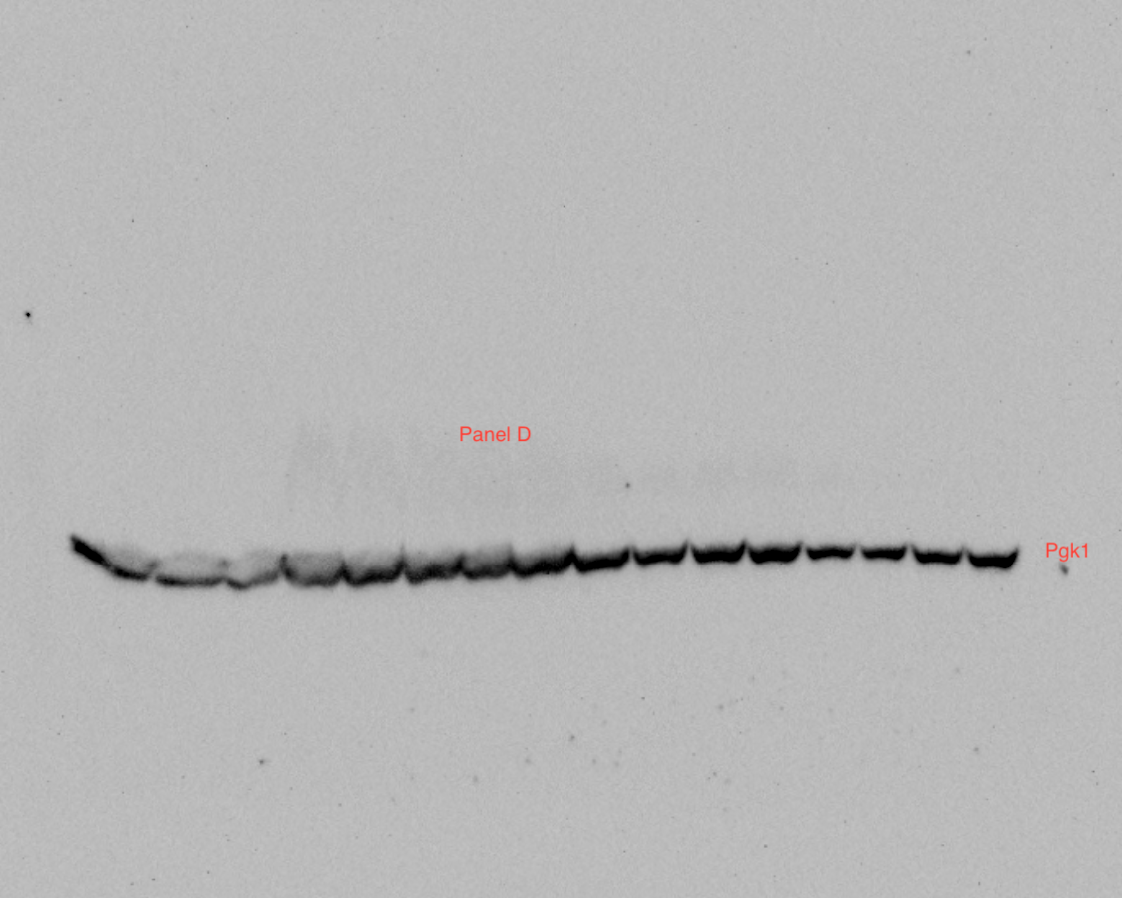

Supplement: Figure 3—figure supplement 1—source data 2. [file elife-110117-fig3-figsupp1-data2.zip › Figure3-figure supplement 1-source data 2/D/Pgk1_annotated.tif]

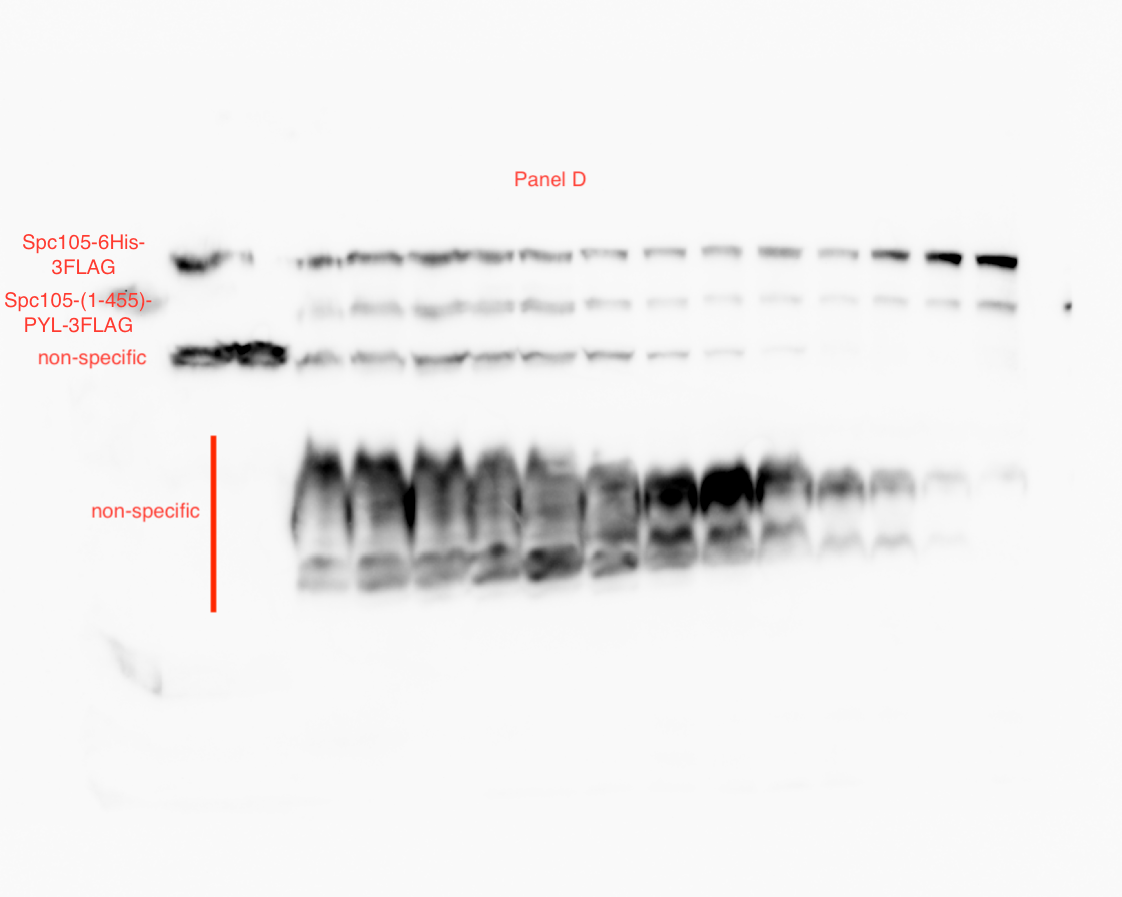

Supplement: Figure 3—figure supplement 1—source data 2. [file elife-110117-fig3-figsupp1-data2.zip › Figure3-figure supplement 1-source data 2/D/FLAG_annotated.tif]

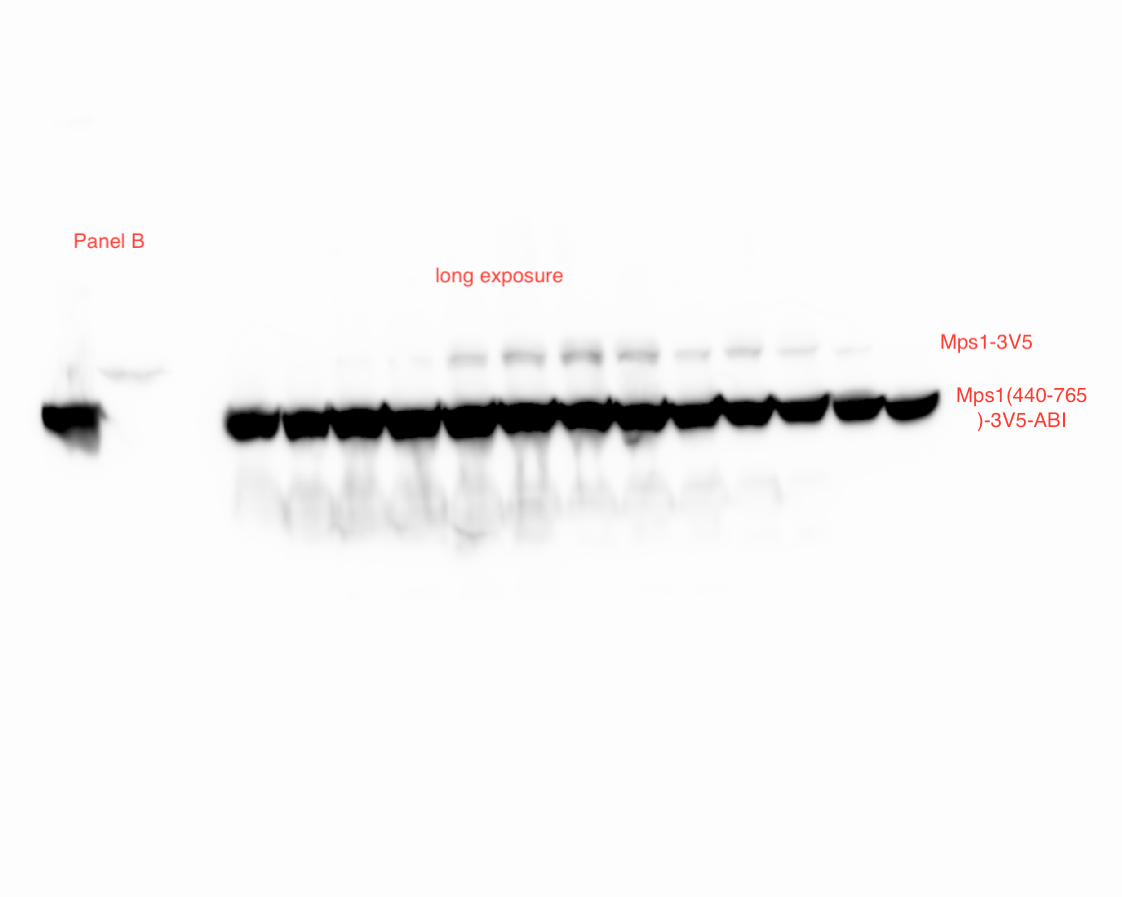

Supplement: Figure 3—figure supplement 1—source data 2. [file elife-110117-fig3-figsupp1-data2.zip › Figure3-figure supplement 1-source data 2/B/V5_long_annotated.tif]

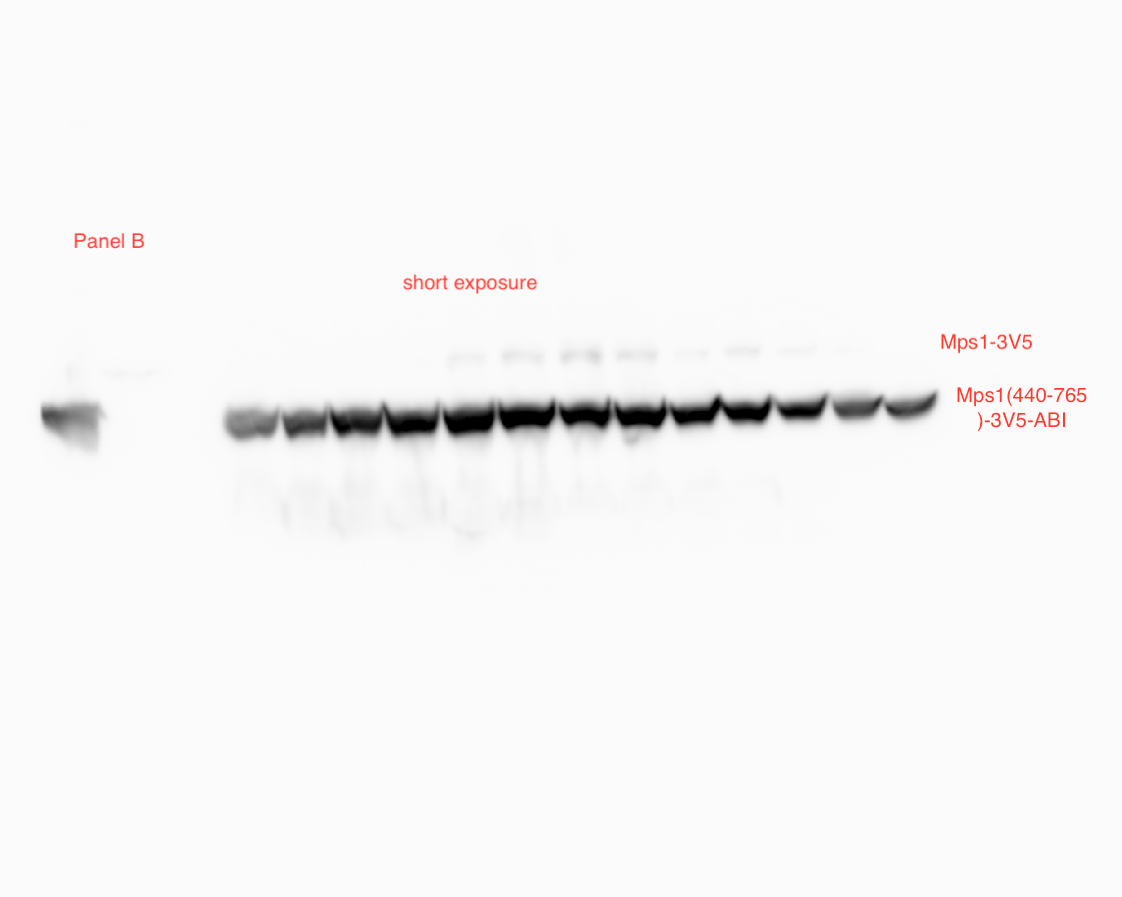

Supplement: Figure 3—figure supplement 1—source data 2. [file elife-110117-fig3-figsupp1-data2.zip › Figure3-figure supplement 1-source data 2/B/V5_short_annotated.tif]

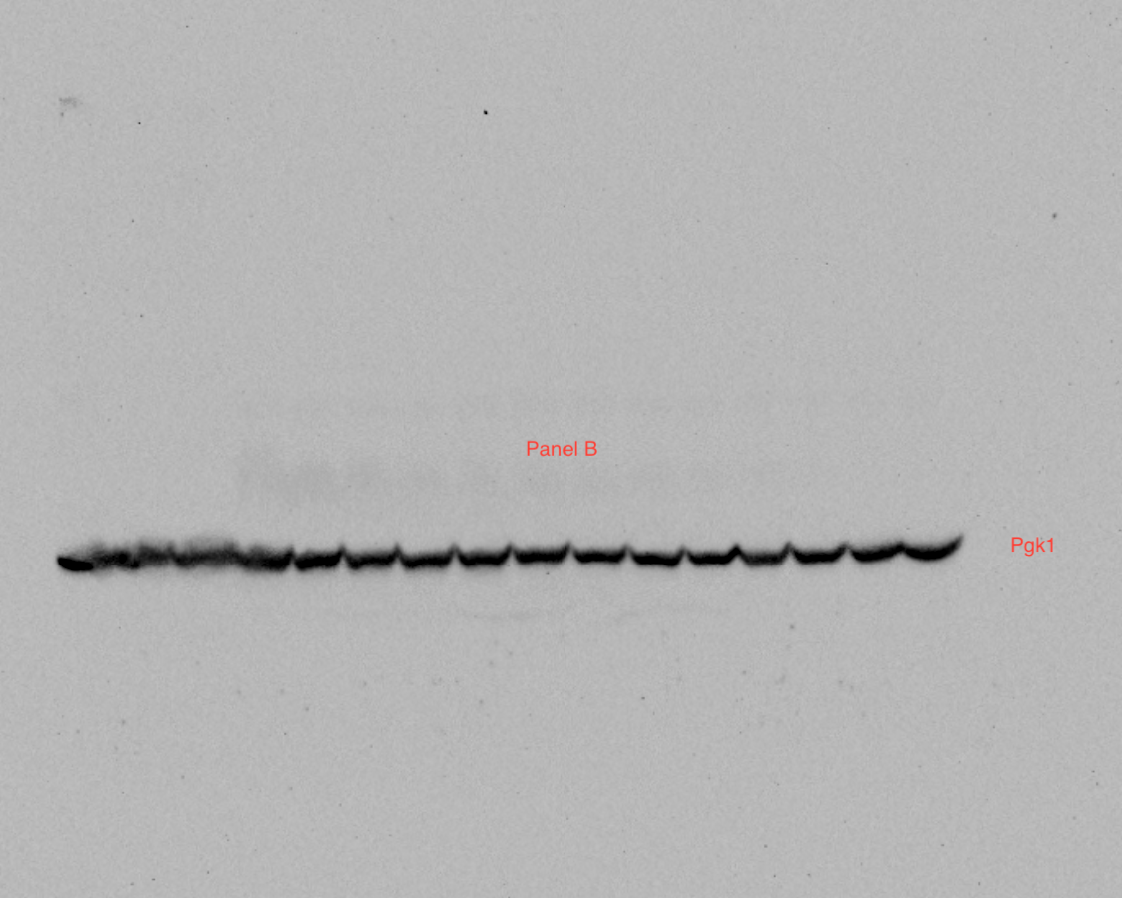

Supplement: Figure 3—figure supplement 1—source data 2. [file elife-110117-fig3-figsupp1-data2.zip › Figure3-figure supplement 1-source data 2/B/Pgk1_annotated.tif]

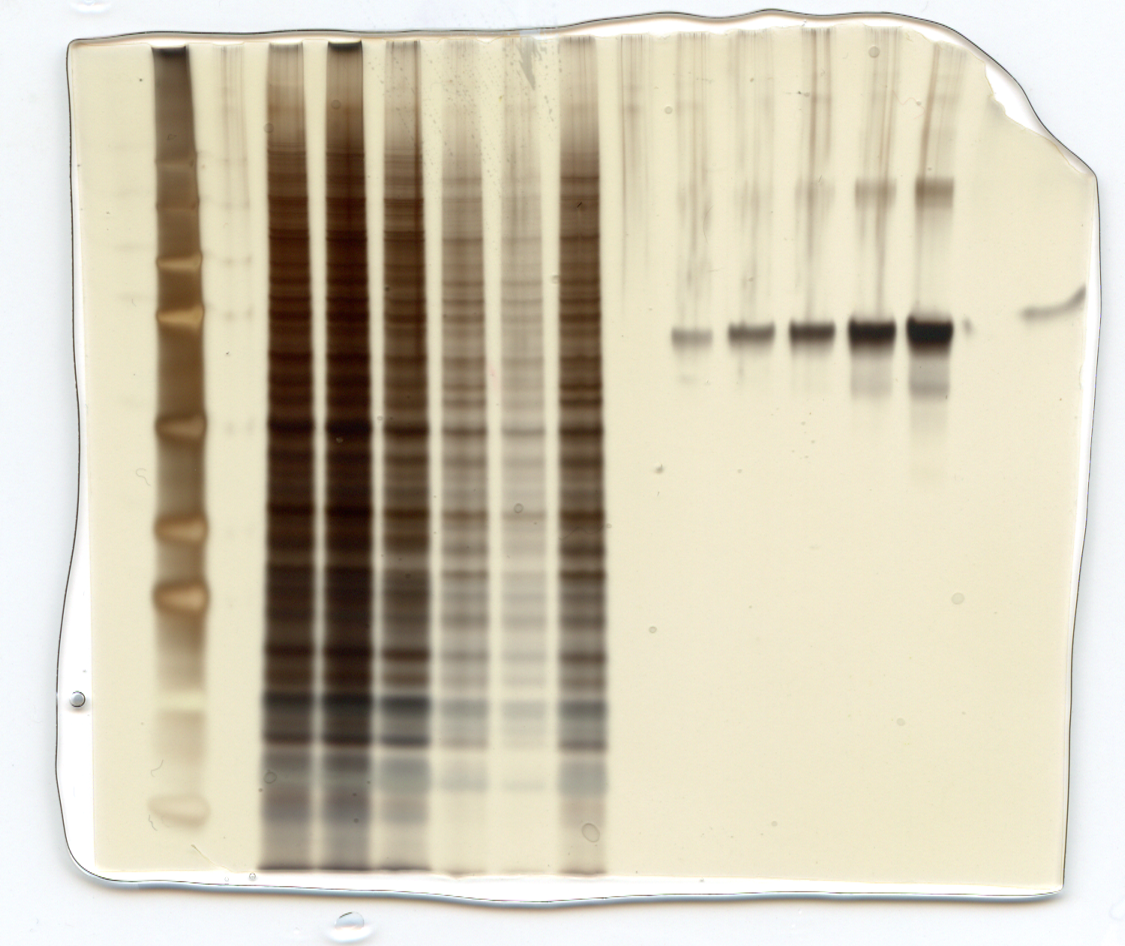

Supplement: Figure 5—figure supplement 1—source data 1. [file elife-110117-fig5-figsupp1-data1.zip › Figure5-figure supplement 1-source data 1/240508_51K_prophase.tif]

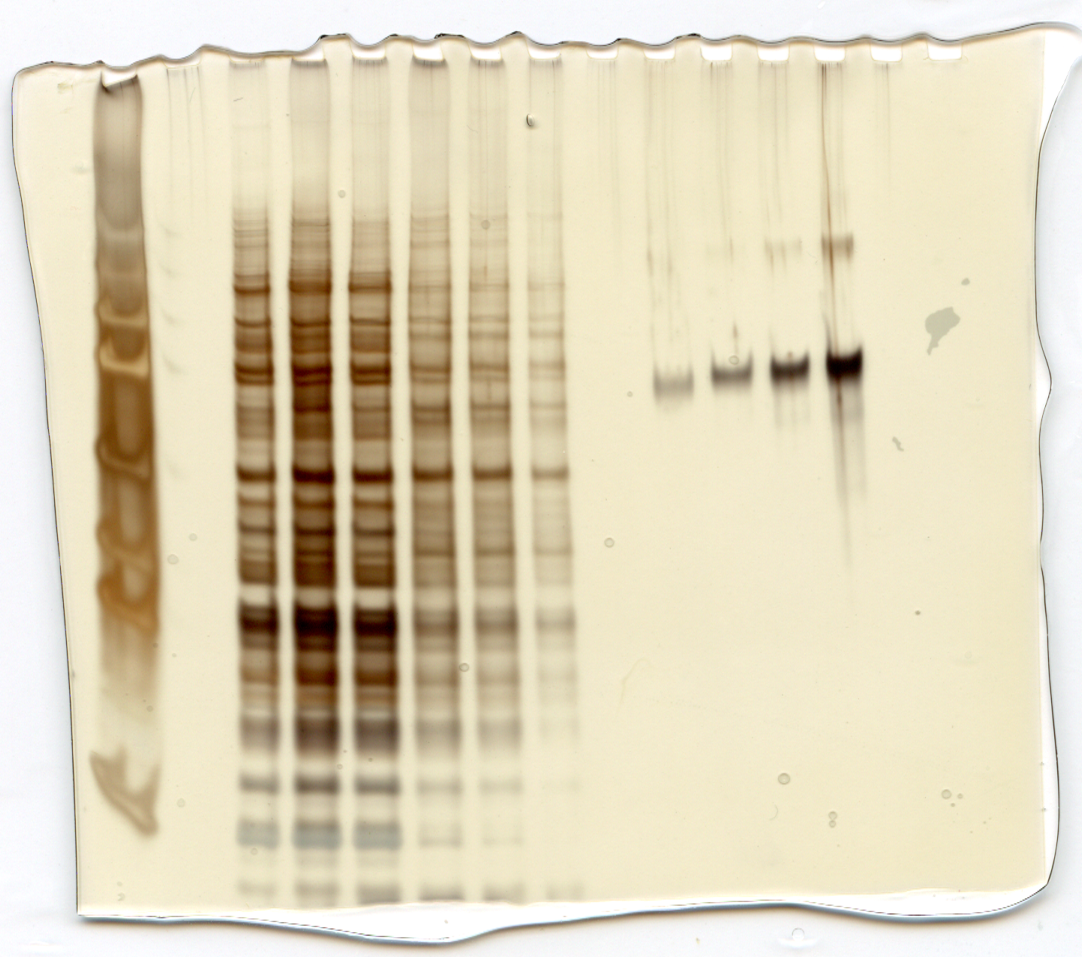

Supplement: Figure 5—figure supplement 1—source data 1. [file elife-110117-fig5-figsupp1-data1.zip › Figure5-figure supplement 1-source data 1/240528_51L_3_metaphase2.tif]

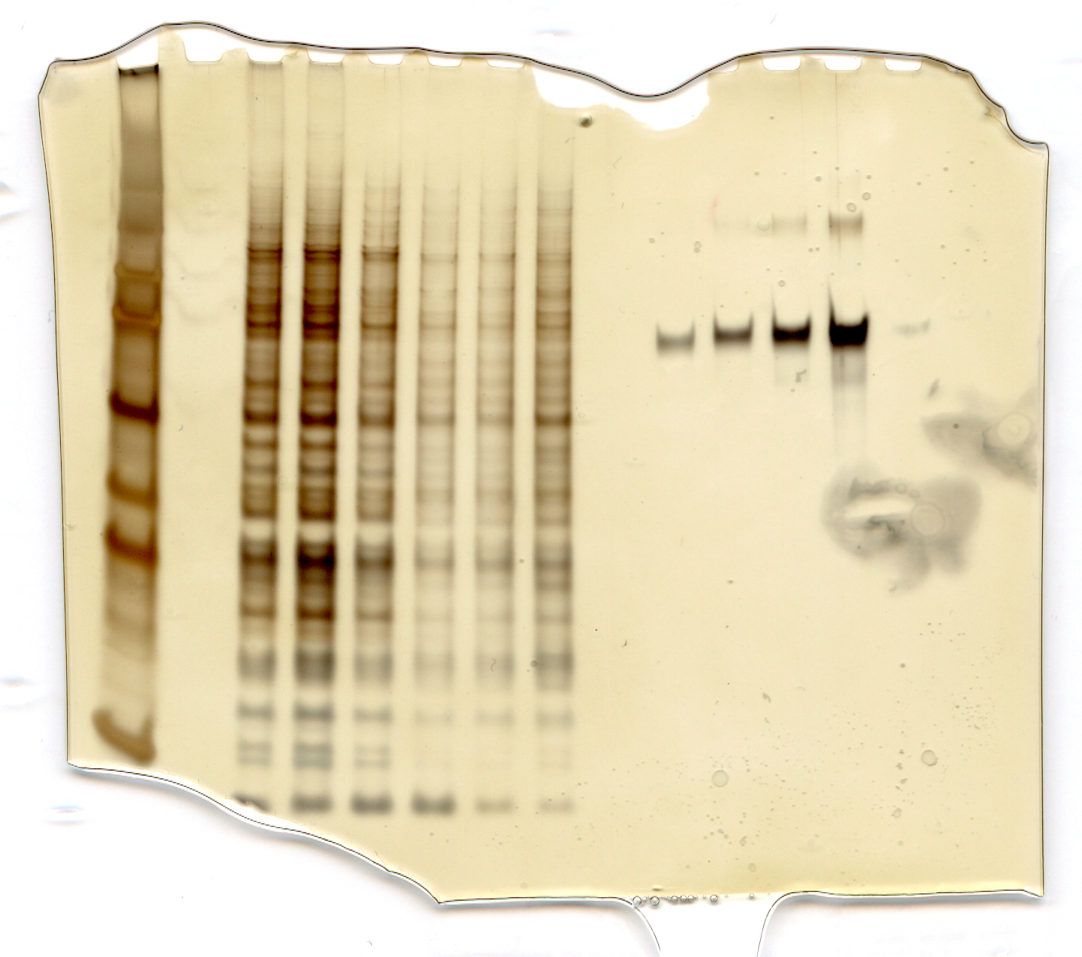

Supplement: Figure 5—figure supplement 1—source data 1. [file elife-110117-fig5-figsupp1-data1.zip › Figure5-figure supplement 1-source data 1/240528_51M_metaphase1.tif]

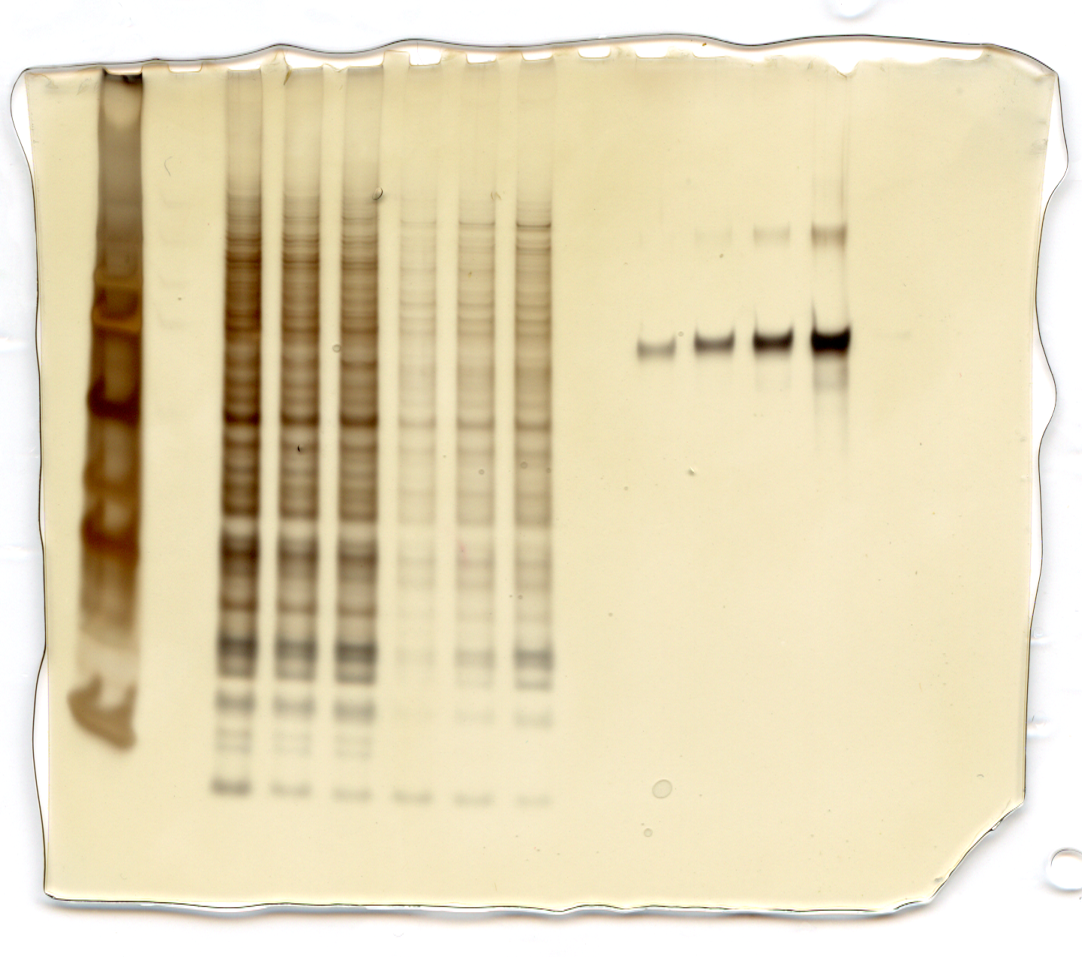

Supplement: Figure 5—figure supplement 1—source data 1. [file elife-110117-fig5-figsupp1-data1.zip › Figure5-figure supplement 1-source data 1/240528_51N_mitotic_metaphase.tif]

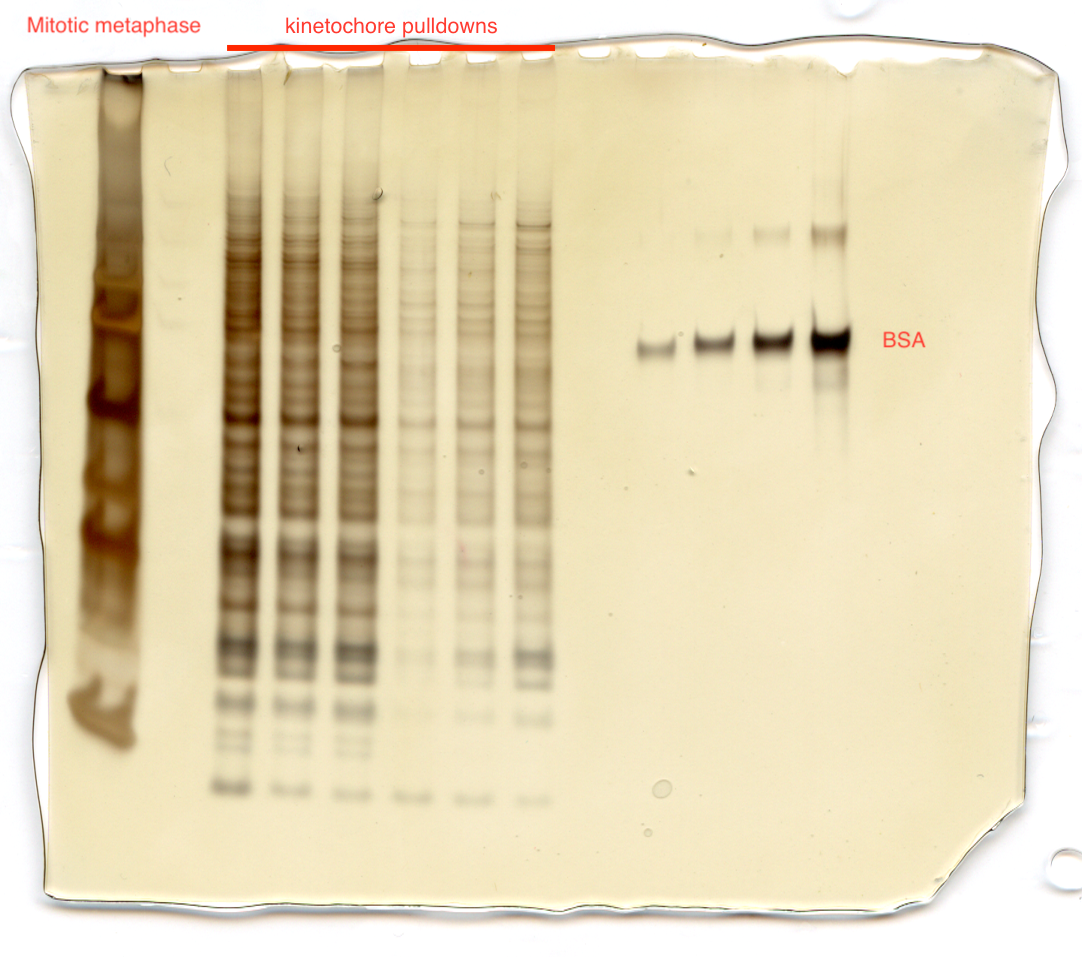

Supplement: Figure 5—figure supplement 1—source data 2. [file elife-110117-fig5-figsupp1-data2.zip › Figure5-figure supplement 1-source data 2/240528_51N_mitotic_metaphase_annotated.tif]

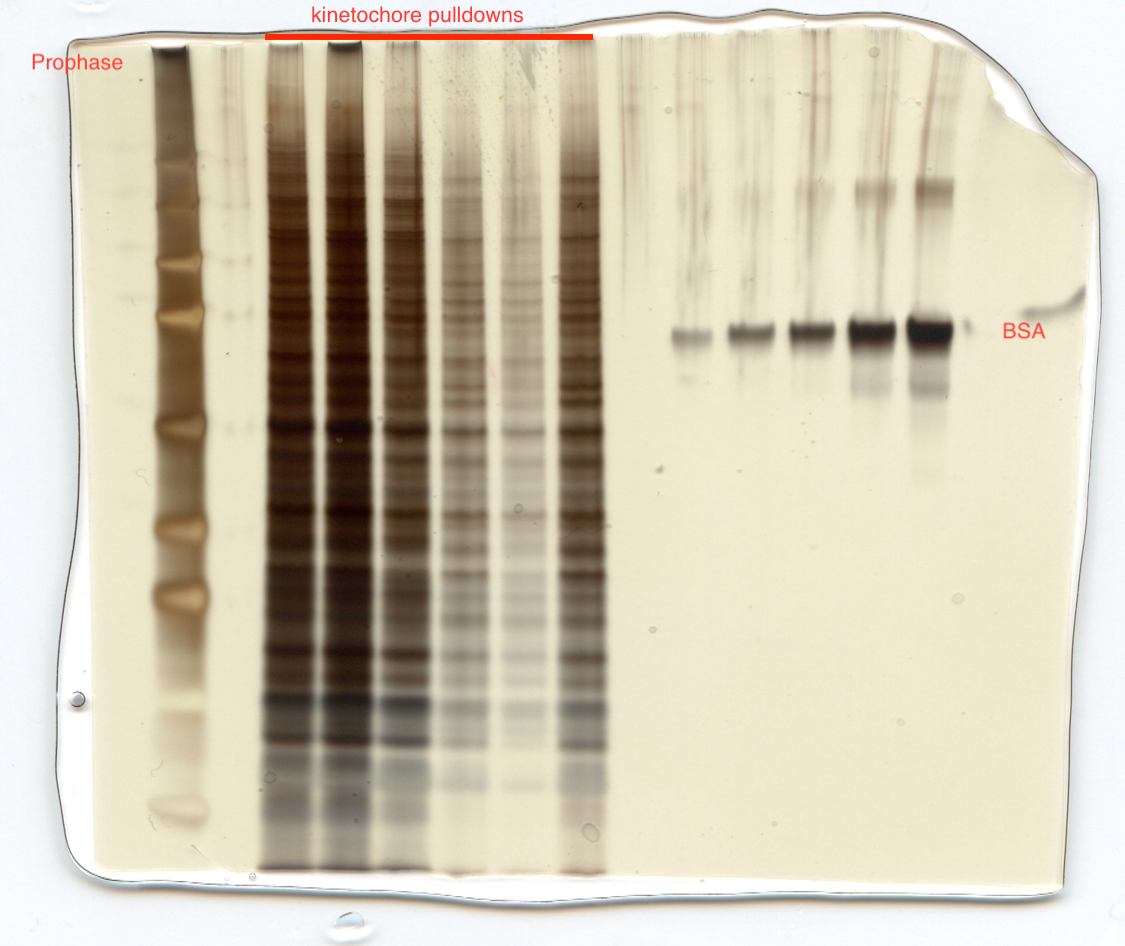

Supplement: Figure 5—figure supplement 1—source data 2. [file elife-110117-fig5-figsupp1-data2.zip › Figure5-figure supplement 1-source data 2/240508_51K_prophase_annotated.tif]

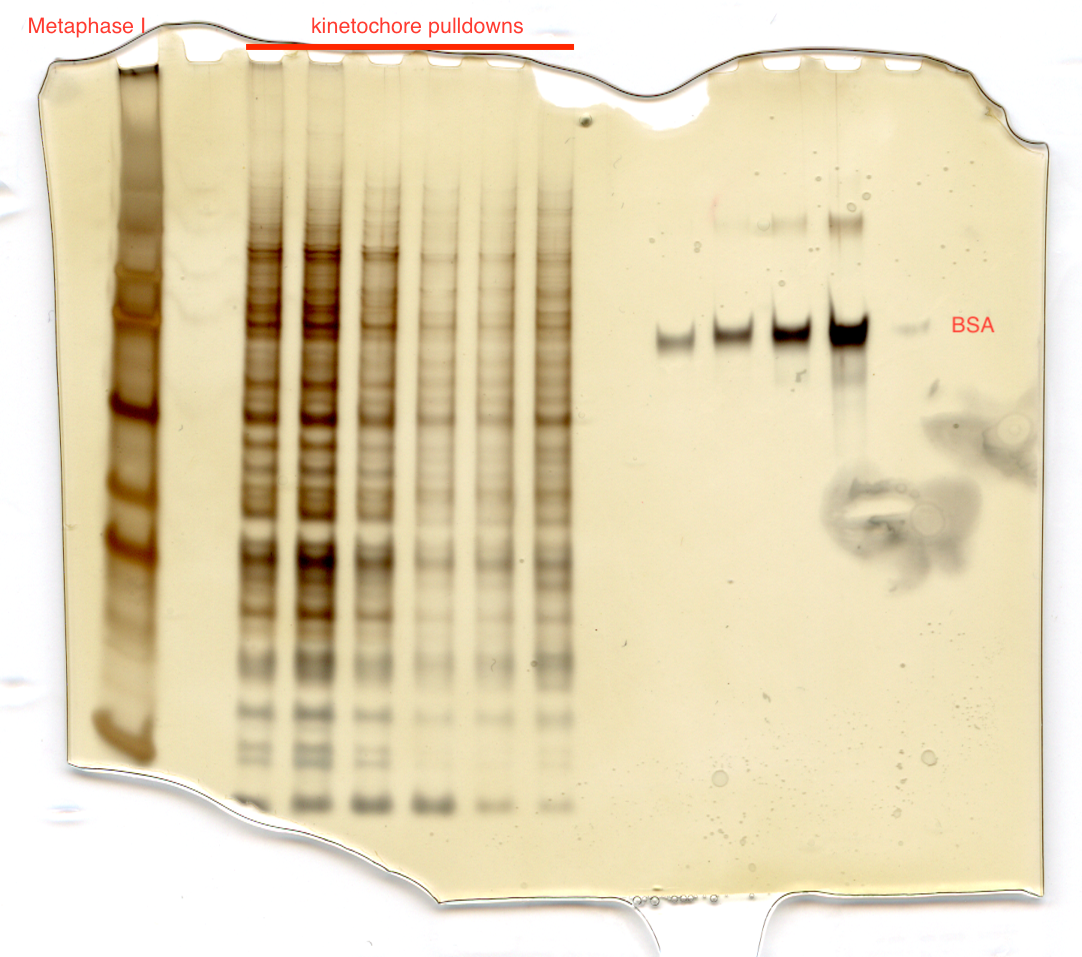

Supplement: Figure 5—figure supplement 1—source data 2. [file elife-110117-fig5-figsupp1-data2.zip › Figure5-figure supplement 1-source data 2/240528_51M_metaphase1_annotated.tif]

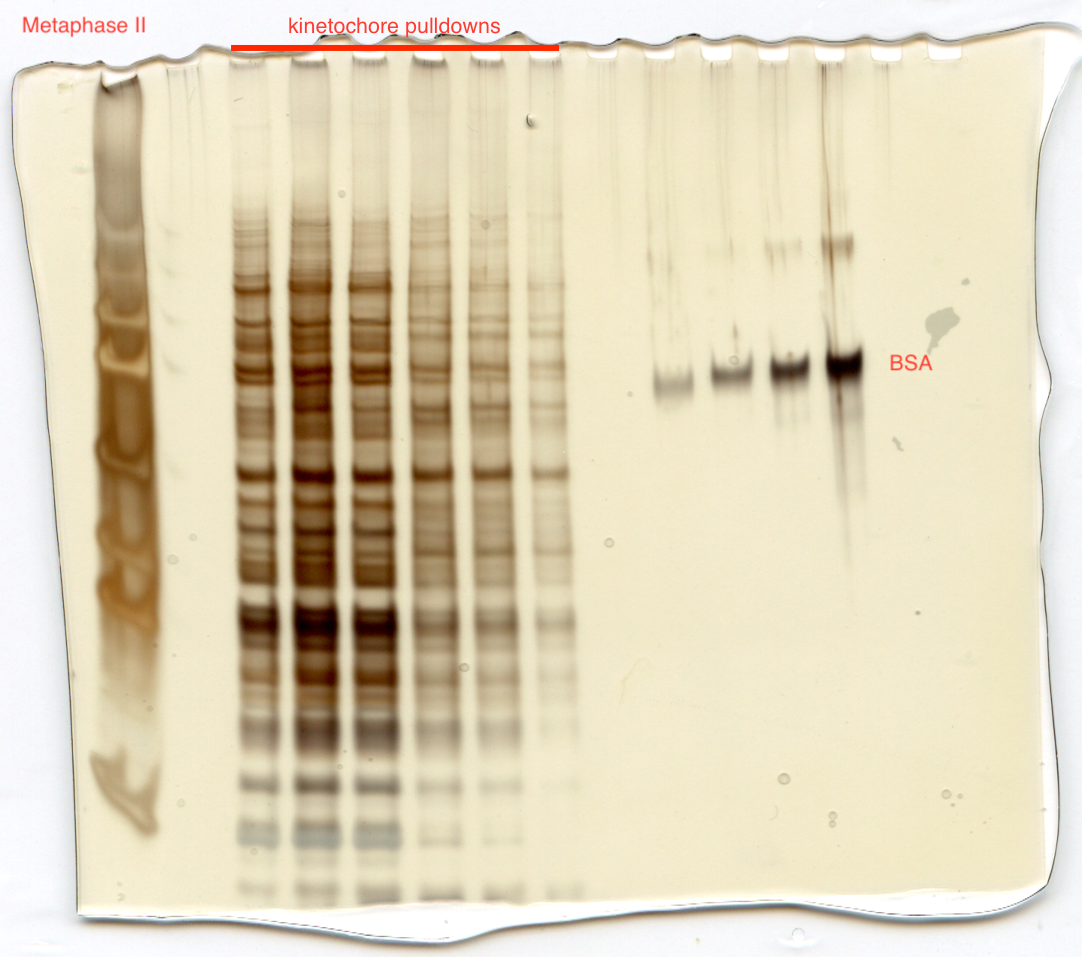

Supplement: Figure 5—figure supplement 1—source data 2. [file elife-110117-fig5-figsupp1-data2.zip › Figure5-figure supplement 1-source data 2/240528_51L_3_metaphase2_annotated.tif]
